# A roadmap of isolating and investigating bacteriophage infecting human gut anaerobes

**DOI:** 10.1042/EBC20240116

**Published:** 2024-12-17

**Authors:** Cong Liu, Bo Xing, Zhuoran Li, Junhua Li, Minfeng Xiao

**Affiliations:** 1 BGI Research, Shenzhen 518083, China; 2 College of Life Sciences, University of Chinese Academy of Sciences, Beijing 100049, China; 3 BGI Research, Belgrade 11000, Serbia; 4 Shenzhen Key Laboratory of Unknown Pathogen Identification, BGI Research, Shenzhen 518083, China

**Keywords:** bacteriophages, human gut bacteria, isolation, obligate anaerobes

## Abstract

Bacteriophages, viruses that infect bacteria, play a crucial role in manipulating the gut microbiome, with implications for human health and disease. Despite the vast amount of data available on the human gut virome, the number of cultured phages that infect human gut bacteria—particularly obligate anaerobes—remains strikingly limited. Here, we summarize the resources and basic characteristics of phages that infect the human gut obligate anaerobe. We review various methods for isolating these phages and suggest a strategy for their isolation. Additionally, we outline their impact on the field of viral biology, their interactions with bacteria and humans, and their potential for disease intervention. Finally, we discuss the value and prospects of research on these phages, providing a comprehensive ‘Roadmap’ that sheds light on the ‘dark matter’ of phages that infect human gut obligate anaerobes.

## Introduction

Bacteriophages are highly abundant and diverse within the human gastrointestinal tract (GIT), playing a crucial role in shaping the structure and stability of the gut microbiota. They hold promising therapeutic potential for chronic disease caused by imbalances in human bacteria. Previously, several comprehensive human gut virome databases have been published [1–4], each containing hundreds of thousands of non-redundant virus genomes [5]. Compared with the vast amount of human gut virome data, research on phage isolates specifically targeting human gut bacteria, especially those that target the predominant obligate anaerobes in the gut, remains sparse—this is not due to a lack of interest, but rather the difficulty in obtaining such phages. Nevertheless, combined with the advanced metagenomics and culturomics technologies, several phages infecting different human gut bacterial taxa have been isolated by separate studies successively [6–16], providing a multitude of representative strategies for phage isolation. Despite these advances, our understanding of phages infecting gut bacteria—particularly those targeting obligate anaerobes—remains limited, and the current strategies have yet to fully overcome the barriers to isolation [1,17]. This knowledge gap has led to a limited number of culturable phages that infect human gut obligate anaerobic bacteria, constraining the full exploration of their potential. To provide a ‘Roadmap’ for research on phages targeting human gut bacteria, we briefly review phage isolates, particularly those targeting human gut obligate anaerobes, summarize available data on the diversity, taxonomy, and basic characteristics of these phages, suggest an approach for phage isolation, and explore their implications. By discussing their value and future perspectives, we hope to illuminate the ‘dark matter’ of the gut microbiota and contribute to the understanding of the virus.

## The phages infect bacteria in the human gut

To comprehensively review phages targeting human gut bacteria, we first cataloged these bacteria from four databases established based on extensive metagenome data sets or isolated biobanks: the Unified Human Gastrointestinal Genome (UHGG) [18], the Culturable Genome Reference (CGR) [19], the Human Gastrointestinal Bacteria Culture Collection (HBC) [20], and human Gut Microbial Biobank (hGMB) [21]. Almeida et al. compiled and analyzed 204,938 genomes and 170,602,708 genes from human gut microbiome data sets to generate the UHGG [18]. Zou et al. presented a reference catalog of genomes of cultivated human gut bacteria (CGR) by collecting 1,520 non-redundant, high-quality draft genomes from over 6,000 bacteria cultivated from fecal samples of healthy humans [19]. Forster et al. presented the HBC, including a comprehensive set of 737 whole-genome-sequenced bacterial isolates, representing 273 species from 31 families [20]. Liu et al. established the hGMB, which contains 1,170 strains and represents 400 human gut microbial species [21]. These four studies comprehensively demonstrate the diversity of human gut bacteria, serving as sources of information for our search to identify host bacteria for targeted phages.

To summarize the proportions of obligate anaerobes and aerobes or facultative anaerobes in the human gut microbiota, as well as the corresponding proportions of isolated phages, we organized the data through the following steps: (1) We conducted a comprehensive review of four human gut bacterial data sets mentioned above, removing redundancies to retain only those with clearly defined taxonomic classifications. This resulted in a dataset of human gut bacteria with clearly defined species classifications, including obligate anaerobes and aerobe or facultative anaerobes. (2) We retrieved information on phage isolates from the NCBI database (updated on April 8, 2024), which included details on the species of targeted host bacteria, isolation sources. (3) We integrated and compared the data from the previous two steps. By cross-referencing the phage isolates’ host bacteria information with our compiled lists of obligate anaerobes and aerobe or facultative anaerobes, we identified the phage isolates that specifically target these two categories of bacteria.

Based on these criteria, we compiled a total of 315 species of human gut bacteria with clearly defined classifications. Among these, the proportion of obligate anaerobes ranged from 61.98% to 93.31%, while the remaining 6.69% to 37.93% were aerobes or facultative anaerobes, indicating that obligate anaerobes pre-dominated in the gut bacterial community (Table 1). Regarding phages, we identified 8,583 phage isolates targeting the aforementioned 315 species of human gut bacteria from the NCBI data base, with the number of phages targeting aerobic or facultative anaerobes (95.50%) significantly exceeding that targeting obligate anaerobes (4.50%) (Table 1). While preliminary, this finding suggests that despite the predominance of obligate anaerobic bacteria among the human gut bacteria community, phage isolates specific to these bacteria are remarkably scarce and underrepresented in research. This may be due to (1) existing isolation methods may not be entirely suitable for isolating phages that infect gut obligate anaerobes, (2) the biological characteristics of these phages remain unclear, and (3) the interaction mechanisms between the phage and their host bacteria have not been elucidated, further complicating the isolation process. Therefore, reviewing phage isolates targeting obligate anaerobes in the human gut is urgent, as it will provide valuable insights into their unique biological properties and interactions with other biological entities. A deeper understanding will advance the knowledge of these phages and improve their isolation, thereby facilitating their application.

**Table 1:** Proportion of human gut bacteria and phage targeting human gut bacteria with different oxygen requirements

	Proportion of human gut bacteria from four data bases with different oxygen requirement								Proportion of phages targeting human gut bacteria with different oxygen requirements	
	Culturable Genome Reference (CGR) [19]		Human Gastrointestinal Bacteria Culture Collection (HBC) [20]		Human Gut Microbial Biobank (hGMB) [21]		Unified Human Gastrointestinal Genome (UHGG) [18]			
	Number	Proportion	Number	Proportion	Number	Proportion	Number	Proportion	Number	Proportion
Obligate anaerobes	1326	93.31%	714	61.98%	8266	78.36%	197969	89.36%	385	4.50%
Aerobes/facultative anaerobes	95	6.69%	437	37.93%	2239	21.22%	22805	10.29%	8198	95.50%
Unknown	0	0.00%	1	0.09%	44	0.42%	763	0.34%	0	0.00%

## Current understanding of phage infecting human gut obligate anaerobes

To delve into phages targeting human gut obligate anaerobes, we selected existing phages that target these anaerobes based on the literature (Table 2). These phages must have corresponding literature references to allow for traceability and review, thereby providing insights into their biological and genomic characteristics and ensuring that their host bacteria are obligate anaerobes isolated from the human gut (Table 2).

**Table 2:** 212 isolated phages targeting human gut obligate anaerobes

Accession	Host bacteria	Bacterial host source	Ref
MH675552	*Bacteroides intestinalis*	Human feces	[42,43]
MN917146	*Bacteroides xylanisolvens*	Human feces	[44]
MN929097	*Parabacteroides distasonis*	Human feces	[30]
MT074134	*Bacteroides thetaiotaomicron*	Human feces	[45]
MT074135	*Bacteroides thetaiotaomicron*	Human feces	
MT074136	*Bacteroides thetaiotaomicron*	Human feces	
MT074137	*Bacteroides thetaiotaomicron*	Human feces	
MT074138	*Bacteroides thetaiotaomicron*	Human feces	
MT074139	*Bacteroides thetaiotaomicron*	Human feces	
MT074140	*Bacteroides thetaiotaomicron*	Human feces	
MT074141	*Bacteroides thetaiotaomicron*	Human feces	
MT074142	*Bacteroides thetaiotaomicron*	Human feces	
MT074143	*Bacteroides thetaiotaomicron*	Human feces	
MT074144	*Bacteroides thetaiotaomicron*	Human feces	
MT074145	*Bacteroides thetaiotaomicron*	Human feces	
MT074146	*Bacteroides thetaiotaomicron*	Human feces	
MT074147	*Bacteroides thetaiotaomicron*	Human feces	
MT074148	*Bacteroides thetaiotaomicron*	Human feces	
MT074149	*Bacteroides thetaiotaomicron*	Human feces	
MT074150	*Bacteroides thetaiotaomicron*	Human feces	
MT074151	*Bacteroides thetaiotaomicron*	Human feces	
MT074152	*Bacteroides thetaiotaomicron*	Human feces	
MT074153	*Bacteroides thetaiotaomicron*	Human feces	
MT074154	*Bacteroides thetaiotaomicron*	Human feces	
MT074155	*Bacteroides thetaiotaomicron*	Human feces	
MT074156	*Bacteroides thetaiotaomicron*	Human feces	
MT074157	*Bacteroides thetaiotaomicron*	Human feces	
MT074158	*Bacteroides thetaiotaomicron*	Human feces	
MT074159	*Bacteroides thetaiotaomicron*	Human feces	
MT074160	*Bacteroides thetaiotaomicron*	Human feces	
MT806185	*Bacteroides uniformis*	Human feces	[46]
MT806187	*Bacteroides uniformis*	Human feces	
MT980836	*Ruminococcus gnavus*	Human feces	[27]
MT980837	*Ruminococcus gnavus*	Human feces	
MT980838	*Ruminococcus gnavus*	Human feces	
MT980839	*Ruminococcus gnavus*	Human feces	
MT980840	*Ruminococcus gnavus*	Human feces	
MT980841	*Ruminococcus gnavus*	Human feces	
MW512570	*Clostridioides difficile*	Human feces	[11]
MW512571	*Clostridioides difficile*	Human feces	
MW512572	*Clostridioides difficile*	Human feces	
MW512573	*Clostridioides difficile*	Human feces	
MW916539	*Bacteroides fragilis*	Human feces	[47]
ON721384	*Bacteroides uniformis*	Human feces	[48]
ON721385	*Bacteroides uniformis*	Human feces	
OP172633	*Agathobaculum butyriciproducens*	Human feces	[16]
OP172634	*Agathobaculum butyriciproducens*	Human feces	
OP172635	*Anaerostipes caccae*	Human feces	
OP172636	*Anaerostipes caccae*	Human feces	
OP172637	*Anaerostipes caccae*	Human feces	
OP172640	*Alistipes shahii*	Human feces	
OP172641	*Bifidobacterium adolescentis*	Human feces	
OP172642	*Bifidobacterium adolescentis*	Human feces	
OP172643	*Bacteroides ovatus*	Human feces	
OP172644	*Bacteroides ovatus*	Human feces	
OP172645	*Bacteroides ovatus*	Human feces	
OP172646	*Bacteroides ovatus*	Human feces	[16]
OP172647	*Bacteroides ovatus*	Human feces	
OP172648	*Bacteroides ovatus*	Human feces	
OP172649	*Bacteroides ovatus*	Human feces	
OP172650	*Bacteroides ovatus*	Human feces	
OP172651	*Bacteroides ovatus*	Human feces	
OP172658	*Bifidobacterium dentium*	Human feces	
OP172659	*Bifidobacterium dentium*	Human feces	
OP172660	*Bifidobacterium dentium*	Human feces	
OP172661	*Bacteroides fragilis*	Human feces	
OP172662	*Bacteroides fragilis*	Human feces	
OP172663	*Bacteroides fragilis*	Human feces	
OP172664	*Bacteroides fragilis*	Human feces	
OP172665	*Bacteroides fragilis*	Human feces	
OP172666	*Bacteroides fragilis*	Human feces	
OP172667	*Bacteroides fragilis*	Human feces	
OP172668	*Bacteroides fragilis*	Human feces	
OP172669	*Bacteroides fragilis*	Human feces	
OP172670	*Bacteroides fragilis*	Human feces	
OP172671	*Bacteroides fragilis*	Human feces	
OP172672	*Bacteroides fragilis*	Human feces	
OP172673	*Bacteroides fragilis*	Human feces	
OP172674	*Bacteroides fragilis*	Human feces	
OP172675	*Bacteroides fragilis*	Human feces	
OP172676	*Bacteroides fragilis*	Human feces	
OP172677	*Bacteroides fragilis*	Human feces	
OP172678	*Bacteroides fragilis*	Human feces	
OP172679	*Bacteroides fragilis*	Human feces	
OP172680	*Bacteroides ovatus*	Human feces	
OP172681	*Bacteroides ovatus*	Human feces	
OP172682	*Bacteroides ovatus*	Human feces	
OP172683	*Bacteroides ovatus*	Human feces	
OP172684	*Bacteroides ovatus*	Human feces	
OP172685	*Bacteroides ovatus*	Human feces	
OP172686	*Bacteroides ovatus*	Human feces	
OP172687	*Bacteroides ovatus*	Human feces	
OP172688	*Bacteroides ovatus*	Human feces	
OP172689	*Bacteroides ovatus*	Human feces	
OP172690	*Bacteroides ovatus*	Human feces	
OP172691	*Bacteroides ovatus*	Human feces	
OP172692	*Bacteroides ovatus*	Human feces	
OP172693	*Bacteroides ovatus*	Human feces	
OP172698	*Bacteroides ovatus*	Human feces	
OP172699	*Bacteroides ovatus*	Human feces	
OP172700	*Bifidobacterium pseudocatenulatum*	Human feces	
OP172701	*Bifidobacterium pseudocatenulatum*	Human feces	
OP172702	*Bifidobacterium pseudocatenulatum*	Human feces	
OP172703	*Bifidobacterium pseudocatenulatum*	Human feces	
OP172704	*Bifidobacterium pseudocatenulatum*	Human feces	
OP172705	*Bifidobacterium pseudocatenulatum*	Human feces	
OP172706	*Bacteroides salyersiae*	Human feces	
OP172707	*Bacteroides salyersiae*	Human feces	
OP172708	*Bacteroides thetaiotaomicron*	Human feces	
OP172709	*Bacteroides thetaiotaomicron*	Human feces	
OP172710	*Bacteroides thetaiotaomicron*	Human feces	
OP172711	*Bacteroides thetaiotaomicron*	Human feces	
OP172712	*Bacteroides thetaiotaomicron*	Human feces	[16]
OP172713	*Bacteroides thetaiotaomicron*	Human feces	
OP172714	*Bacteroides thetaiotaomicron*	Human feces	
OP172715	*Bacteroides thetaiotaomicron*	Human feces	
OP172716	*Bacteroides thetaiotaomicron*	Human feces	
OP172717	*Bacteroides thetaiotaomicron*	Human feces	
OP172718	*Bacteroides thetaiotaomicron*	Human feces	
OP172719	*Bacteroides thetaiotaomicron*	Human feces	
OP172720	*Bacteroides thetaiotaomicron*	Human feces	
OP172721	*Bacteroides thetaiotaomicron*	Human feces	
OP172722	*Bacteroides thetaiotaomicron*	Human feces	
OP172723	*Bacteroides thetaiotaomicron*	Human feces	
OP172724	*Bacteroides thetaiotaomicron*	Human feces	
OP172725	*Bacteroides thetaiotaomicron*	Human feces	
OP172726	*Bacteroides thetaiotaomicron*	Human feces	
OP172727	*Bacteroides thetaiotaomicron*	Human feces	
OP172728	*Bacteroides thetaiotaomicron*	Human feces	
OP172729	*Bacteroides thetaiotaomicron*	Human feces	
OP172730	*Bacteroides thetaiotaomicron*	Human feces	
OP172731	*Bacteroides uniformis*	Human feces	
OP172732	*Bacteroides uniformis*	Human feces	
OP172733	*Bacteroides uniformis*	Human feces	
OP172734	*Bacteroides uniformis*	Human feces	
OP172735	*Bacteroides uniformis*	Human feces	
OP172751	*Bacteroides xylanisolvens*	Human feces	
OP172752	*Clostridium butyricum*	Human feces	
OP172758	*Hungatella sp005845265*	Human feces	
OP172759	*Clostridium sp003481775*	Human feces	
OP172760	*Clostridium sp003481775*	Human feces	
OP172761	*Clostridium innocuum*	Human feces	
OP172762	*Clostridium innocuum*	Human feces	
OP172763	*Clostridium sp003481775*	Human feces	
OP172764	*Clostridium_sp003481775*	Human feces	
OP172765	*Clostridium sp003481775*	Human feces	
OP172766	*Clostridium innocuum*	Human feces	
OP172767	*Clostridium innocuum*	Human feces	
OP172768	*Clostridium innocuum*	Human feces	
OP172769	*Clostridium innocuum*	Human feces	
OP172770	*Clostridium innocuum*	Human feces	
OP172771	*Clostridium innocuum*	Human feces	
OP172772	*Clostridium symbiosum*	Human feces	
OP172773	*Clostridium symbiosum*	Human feces	
OP172774	*Clostridium symbiosum*	Human feces	
OP172775	*Clostridium sp000509125*	Human feces	
OP172776	*Clostridium sp000509125*	Human feces	
OP172777	*Clostridium sp000509125*	Human feces	
OP172778	*Clostridium sp000509125*	Human feces	
OP172779	*Dorea sp. 000433215*	Human feces	
OP172808	*Eggerthella lenta*	Human feces	
OP172814	*Parabacteroides distasonis*	Human feces	
OP172815	*Parabacteroides distasonis*	Human feces	[16]
OP172816	*Parabacteroides distasonis*	Human feces	
OP172817	*Parabacteroides distasonis*	Human feces	
OP172818	*Parabacteroides distasonis*	Human feces	
OP172819	*Parabacteroides faecis*	Human feces	
OP172820	*Parabacteroides faecis*	Human feces	
OP172821	*Parabacteroides merdae*	Human feces	
OP172822	*Parabacteroides merdae*	Human feces	
OP172823	*Parabacteroides merdae*	Human feces	
OP172824	*Parabacteroides merdae*	Human feces	
OP172825	*Parabacteroides merdae*	Human feces	
OP172826	*Parabacteroides merdae*	Human feces	
OP172827	*Parabacteroides merdae*	Human feces	
OP172828	*Parabacteroides merdae*	Human feces	
OP172829	*Parabacteroides merdae*	Human feces	
OP172830	*Parabacteroides merdae*	Human feces	
OP172831	*Parabacteroides merdae*	Human feces	
OP172832	*Parabacteroides merdae*	Human feces	
OP172833	*Parabacteroides merdae*	Human feces	
OP172834	*Parabacteroides merdae*	Human feces	
OP172835	*Parabacteroides merdae*	Human feces	
OQ221536	*Bacteroides intestinalis*	Human feces	[29]
OQ221537	*Bacteroides intestinalis*	Human feces	
OQ221538	*Bacteroides intestinalis*	Human feces	
OQ221539	*Bacteroides intestinalis*	Human feces	
OQ221540	*Bacteroides intestinalis*	Human feces	
OQ221541	*Bacteroides intestinalis*	Human feces	
OQ221542	*Bacteroides intestinalis*	Human feces	
OQ221543	*Bacteroides intestinalis*	Human feces	
OQ221544	*Bacteroides intestinalis*	Human feces	
OQ221545	*Bacteroides intestinalis*	Human feces	
OQ221546	*Bacteroides intestinalis*	Human feces	
OQ221547	*Bacteroides intestinalis*	Human feces	
OQ221548	*Bacteroides intestinalis*	Human feces	
OQ221549	*Bacteroides intestinalis*	Human feces	
OQ221550	*Bacteroides intestinalis*	Human feces	
OQ221551	*Bacteroides intestinalis*	Human feces	
OQ221552	*Bacteroides intestinalis*	Human feces	
OQ221553	*Bacteroides intestinalis*	Human feces	
OQ221554	*Bacteroides intestinalis*	Human feces	
OQ221555	*Bacteroides intestinalis*	Human feces	
OQ221556	*Bacteroides intestinalis*	Human feces	
OQ221557	*Bacteroides intestinalis*	Human feces	
OQ221558	*Bacteroides intestinalis*	Human feces	
OQ221559	*Bacteroides intestinalis*	Human feces	
OQ221560	*Bacteroides intestinalis*	Human feces	
OR296437	*Bacteroides uniformis*	Human feces	[49]
OR296438	*Bacteroides uniformis*	Human feces	
OR296439	*Bacteroides uniformis*	Human feces	
OR574845	*Clostridium scindens*	Human feces	[50]

**Figure 1:**
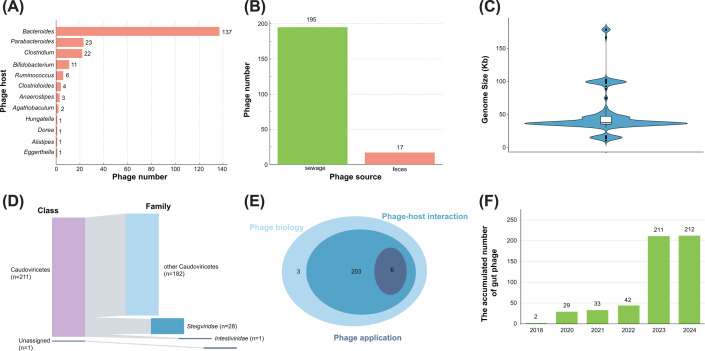
Characteristics of phage isolates targeting human gut obligate anaerobic bacteria and corresponding articles **(A)** The hosts of phage isolates targeting human gut obligate anaerobes. The numbers represent the quantity of the corresponding phages. **(B)** The isolation sources of phage isolates. The different colors correspond to each source category. The numbers represent the quantity of the corresponding phages. **(C)** The distribution of genome sizes of phage isolates. The black bar in the center represents the interquartile range, with the white dot showing the median genome size. The width of each section of the plot indicates the density of phages with that genome size. **(D)** The taxonomy of phage isolates. The different colors correspond to different phage classes and phage families. The numbers represent the quantity of the corresponding phages. **(E)** The Venn diagram illustrating the overlap of research articles focusing on three aspects of phage studies: phage biology, phage-host interactions, and phage applications. The numbers represent the quantity of the corresponding phages. **(F)** The accumulated number of phage isolates infecting human gut anaerobes from 2005 to 2024. The *x*-axis represents the years, while the *y*-axis shows the cumulative number of the phages.

Through this process, we collected a total of 212 phage isolates, of which 137 infect *Bacteroides*, 23 infect *Parabacteroides*, 22 infect *Clostridium*, and 11 infect *Bifidobacterium*, while the remaining isolates infect bacterial genera with fewer than 10 isolates (Figure 1A). Notably, highly abundant bacterial genera in the gut, which host over 10 phage isolates, are well-represented across three biobanks, indicating that the richness of the host bacterial genus is essential for isolating phages. However, some high-abundance bacteria have few associated phages. For example, *Ruminococcus* and *Alistipes*, accounting for 4.08% and 3.80% of the UHGG database, respectively, have only 6 and 1 phage isolates. This discrepancy may be attributed to the unique life cycle of such phages or distinct defense mechanisms carried by these obligate anaerobes against phage infection. Interestingly, despite the high viral diversity targeting Prevotella/Segatella in the viral database [1], no phages infecting these bacteria have been isolated to date [22]. These phages are coined ‘Lak megaphages’ due to their large genome size [17], which may be one of the reasons for their difficulty in isolation, as larger phages are challenging to isolate through plaque assays [17,23]. Furthermore, most phages originate from sewage (195 isolates), while notably, only 17 phages infecting human gut obligate anaerobes have been isolated from feces, indicating a significant gap in exploring and isolating these phages from feces (Figure 1B). The limited recovery from feces could be due to the complexity of the gut environment or the presence of phages that are not easily cultivable under standard laboratory conditions.

Furthermore, based on relevant literature (Table 2), we reviewed the basic characteristics of these phages, including their genome sizes and taxonomic classifications. These summaries are based on information provided in the literature. The genome sizes of these phages range from 10,891 bp to 179,283 bp, with an average size of 49,477 bp (Figure 1C). Among them, except for one genome lacking classification information, all others belong to the class Caudoviricetes, including 28 strains in the family *Steigviridae*, 1 in *Intestiviridae*, and 182 unassigned genomes (Figure 1D). This indicates the diversity of phages targeting obligate anaerobic bacteria and highlights the presence of ‘dark matter’ [24]. Additionally, existing publications on these phages have primarily focused on their biological characteristics, the interaction between phage and host bacteria, and their applications (Figure 1E). Among these topics, ‘phage applications’ have been the least studied. By continuously accumulating resources of phages that infect human gut obligate anaerobic bacteria and expanding our understanding of their interactions with host bacteria and humans, the potential applications of these phages are expected to be significantly enhanced.

Since the first bacteriophage targeting obligate anaerobic gut bacteria was isolated in 2018, a slight increase was observed in 2020. Subsequently, approximately five new phage isolates were discovered each year from 2020 to 2023, with a particularly rapid increase in numbers observed in 2023 (Figure 1F). This trend indicates a growing interest and progress in the study of phages targeting obligate anaerobes, highlighting their importance in gut microbiome research and potential applications. Nonetheless, further exploration is necessary to fully understand the diversity, functionality, and therapeutic potential of bacteriophages targeting obligate anaerobes.

## The approaches for isolating phages infecting gut bacteria

The procedures employed for phage isolation in publications are diverse. In summary, these methods can be categorized into three principal approaches: the classical method, the enrichment method, and the fermentation method. Each approach typically involves three steps: sample pretreatment, enrichment co-culture, and phage screening/isolation.

**Figure 2:**
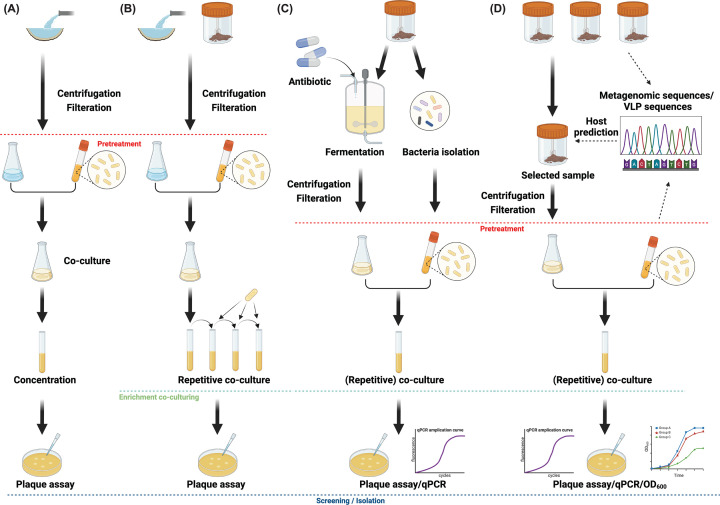
Strategies for precisely isolating phages targeting gut bacteria **(A)** The classical method. **(B)** The enrichment method. **(C)** The fermentation method. **(D)** The metagenomic analysis-based method. Above the red dashed line is the pre-treatment step. Above the green dashed line is the enrichment co-culturing step. Above the dark blue dashed line is the screening/isolation step. The image was created using BioRender.com.

The classical method, widely utilized for isolating phages targeting ESKAPE (*Enterococcus spp., Staphylococcus aureus, Klebsiella pneumoniae, Acinetobacter baumannii, Pseudomonas aeruginosa, and Enterobacter spp*.) pathogens or lactic acid bacteria (LAB) [25,26], is particularly suitable for processing large-volume environmental samples. This method involves two main steps: pretreatment and phage isolation (Figure 2A). First, in the pretreatment step, the original source samples should be centrifuged and filtered to obtain virus-like particles (VLPs) [27]. The VLPs are then co-cultured with the targeted bacterial strains. After propagating for a few hours, the co-culture needs to be centrifuged, filtered, and concentrated to increase the titer of phages [16]. Finally, a plaque assay will be used in the isolation step.

The enrichment method involves all three steps. This method shares similarities with the classical method in the sample pretreatment step but diverges in subsequent steps, notably in the enrichment assay(Figure 2B). There is variability in the enrichment assays across different studies as well, for instance, some studies opt for a single round of enrichment [8], while others employ repetitive enrichment to achieve a higher concentration of the target phages [28,29]. These two methods are not only applicable to isolating phages that infect gut obligate anaerobic bacteria, but also extend to isolating other types of phages that are typically challenging to obtain.

The fermentation method exhibits unique applicability in isolating phages from fecal samples [7,30] (Figure 2C). During fermentation, anoxic conditions are employed, supplemented by antibiotics to suppress the growth of non-targeted bacteria while promoting the proliferation of targeted bacteria. This approach enhances the chance of phage encountering and infecting their host bacteria, facilitating their *in situ* replication. Simultaneously, the bacteria used for phage isolation are derived from the same fecal sample used for fermentation, contributing to the specific targeting of phages. Additionally, the following enrichment co-culture assay is needed to increase the abundance of targeted phages. The phage screening/isolation step utilizes either a traditional plaque assay or q-PCR detection, especially for phages that do not form plaques, such as the crAss002 phage [7]. This method maximizes the preservation of target phages and their host bacteria within the samples, thus enhancing the efficiency of isolating highly specific phages. Concurrently, conducting q-PCR detection on phages that cannot form plaques expands the opportunities for screening phages.

These three established methods isolate phages directly from the samples without prior identification of the microbiome composition, resulting in a blind isolation process and yielding random outcomes. Recently, Ramos-Barbero et al. [29] employed qPCR assays to detect the presence of specific phages with known genome data, such as crAss-like phages, in resource samples. This strategy enables the selection of optimal samples for subsequent phage screening [29], thereby enhancing isolation efficiency. However, it may not be suitable for the discovery of previously unknown phages. With the advancement of sequencing technologies and the decreasing costs associated, researchers have attempted to conduct metagenomic sequencing before phage isolation, using the analysis of the metagenome data to identify phage sequences and predict their bacterial hosts [6]. It provides insight that this approach could be applied to the isolation of phages targeting human gut bacteria. Particularly, as an increasing number of human cohorts have been established, resulting in a growing number of fecal samples with metagenomic data. For these fecal samples containing metagenomic data, we suggest a method that integrates bioinformatics analysis with selected strategies from the aforementioned three methods to achieve the precise isolation of phage targeting specific host bacteria from these samples (Figure 2D). In this method, (1) the host bacteria of viral gene fragments in the samples are predicted through metagenomic data analysis [6]. (2) The relative abundance of phages targeting the specific host bacteria in different samples is analyzed, and the ‘best sample’ with the highest relative abundance of phages targeting the desired host bacteria is identified. (3) These ‘best samples’ are co-cultured with the target host bacterial strains [28], and (4) plaque assays or growth curve analysis and q-PCR techniques can be utilized to validate the success of phage isolation [7,30]. This method effectively achieves precise screening of phages while significantly saving labor and sample resources.

## The implication of phage isolates targeting human gut bacteria

Isolation of phages targeting human gut bacteria provides valuable insights into their characteristics, dynamic interplay between the microbiota and humans, bacterial inhibition, and the potential for disease intervention. Among these phages, crAss-like phages are the most abundant family in the gut and have been studied for their biological properties after isolation [31,32]. By obtaining isolates, researchers can verify their morphology and host specificity [7,28]. Furthermore, the interactions between crAss-like phages and their hosts have been explored, shedding light on the potential mechanisms underlying their long-term persistence in the gut alongside host bacteria [14]. For instance, gut bacteria may use invertible promoters to mediate rapid phase variation of alternate capsular polysaccharides, thereby maintaining a dynamic equilibrium between phage sensitivity and resistance [8,14,33]. However, our current understanding of various elements in crAss-like phages and their interactions with hosts is limited to a small subset of phages. Thus, further screening and exploration of phage isolates are warranted to expand our knowledge in this field.

Phages play a crucial role not only in shaping the human gut microbiota but also in bypassing mammalian physical barriers, influencing human health by directly or indirectly interacting with human cells and the immune system [34,35]. Through predation, phages affect the bacterial host and also affect non-host bacteria through cascading effects mediated by interbacterial interactions, thereby contributing to the architecture of the gut microbiota and influencing the composition of gut metabolites [36]. Moreover, phages can embed themselves within the mucus layer to regulate invasive bacteria, maintaining the integrity of the intestinal barrier [37]. Furthermore, phages can be internalized by epithelial cells and transported to the opposite side, where they release active phages [38]. They also stimulate immune responses by triggering the production of interferon-gamma (IFN-γ) via Toll-like receptor 9 (TLR9), potentially exacerbating intestinal inflammation and colitis [39]. Phages have also been found to impact bacterial phase variation and regulate the levels of regulatory T cells (Tregs), highlighting that phage and host inflammation can drive the dynamic bacterial phase variations [40]. Understanding the dynamic interactions between bacteriophages and gut bacteria offers novel insights into the manipulation of the gut microbiota and the development of targeted therapies.

Additionally, due to their inhibitory properties and specific targeting, phages have the potential to intervene in human chronic disease. Recently, phages of anaerobes, such as *Ruminococcus* phages, have been utilized to treat age-related cognitive dysfunction and have shown potential for treating inflammatory bowel disease (IBD) [13,27]. Furthermore, researchers have successfully modified bacteriophages to carry antimicrobial factors or specific medications. By capitalizing on the targeted capabilities of bacteriophages, these phages can precisely reach the lesions of colorectal cancer (CRC), effectively intervening in the disease progression. It is noteworthy that the modified bacteriophages, carrying drugs, not only deliver the medications directly to the tumor site but also employ the bacteriophage’s antimicrobial properties to reduce the population of *Fusobacterium nucleatum*, a bacterium associated with CRC. As a result, this innovative approach significantly enhances the efficacy of chemotherapy treatments for CRC [12,41].

## Perspectives on phage isolates targeting human gut bacteria

In conclusion, we reviewed the isolation, characteristics, and applications of phage isolates infecting human gut obligate anaerobes and suggested a strategy for phage isolation. It is important to note that the isolation of these phages is merely the first step in phage applications. As the number of phage isolates continues to increase, they will accumulate and supplement the genomic information of gut virome, enhancing the accuracy of viral sequence prediction tools. This will enable us to predict the various phage isolates present in different samples and selectively target and screen phage isolates, thereby effectively improving the efficiency of phage isolation. Furthermore, the phage isolates will increase the genomic diversity of phages and impart additional biological characteristics to the phage, thus elucidating the viral ‘dark matter’, promoting further exploration of the interactions between phages and bacteria *in vitro* and *in vivo*, and confirming the causal relationships between gut bacteria and diseases. Additionally, to expand the applications of phages infecting gut bacteria, we can engineer phages to possess tunable host ranges, design and synthesize novel phages and express phage-derived functional proteins for purposes of host recognition, binding, or lysis. These insights are essential for developing targeted therapeutic approaches and interventions, and they will also expand the boundaries of the phage applications.

## Summary

- Bacteriophages play a crucial role in maintaining the balance of the gut microbiome and have significant implications for human health and disease.
- Despite extensive virome data in the gut, the limited number of culturable phages, especially those targeting human gut obligate anaerobes, constrains a comprehensive exploration of their potential functions.
- We reviewed the phage isolates infecting human gut obligate anaerobes, summarized their characteristics and applications, discussed phage isolation methods, and suggested an approach based on the existing methods to improve isolation efficiency.
- The isolation and research of phages targeting human gut obligate anaerobic bacteria contribute to elucidating their interactions with host bacteria, providing new perspectives for disease intervention.

## Abbreviations

CGR: Culturable Genome Reference
CRC: colorectal cancer
ESKAPE: *Enterococcus spp., Staphylococcus aureus, Klebsiella pneumoniae, Acinetobacter baumannii, Pseudomonas aeruginosa, and Enterobacter spp*.
GIT: gastrointestinal tract
HBC: Human Gastrointestinal Bacteria Culture Collection
hGMB: human Gut Microbial Biobank
IBD: inflammatory bowel disease
IFN-γ: interferon-gamma
LAB: lactic acid bacteria
TLR9: Toll-like receptor 9
UHGG: Unified Human Gastrointestinal Genome
VLP: virus-like particle
